# Biomechanical comparison of static and dynamic cervical plates in terms of the bone fusion, tissue degeneration, and implant behavior

**DOI:** 10.1186/s13018-024-04629-8

**Published:** 2024-02-15

**Authors:** Tzu-Tsao Chung, Dueng-Yuan Hueng, Shang-Chih Lin

**Affiliations:** 1 Graduate Institute of Applied Science and Technology, National Taiwan University of Science and Technology, Taipei, Taiwan; 2 Department of Neurological Surgery, Tri-Service General Hospital, National Defense Medical Center, Taipei, Taiwan; 3 Graduate Institute of Biomedical Engineering, National Taiwan University of Science and Technology, 43 Keelung Road, Section 4, Taipei, 10607 Taiwan

**Keywords:** Dynamic plate, ACDF, ASD, Finite-element analysis, Cervical degeneration

## Abstract

**Introduction:**

Using an anterior cervical fixation device in the anterior cervical discectomy and fusion (ACDF) has evolved to various systems of static and dynamic cervical plates (SCP and DCP). Dynamic cervical plates have been divided into three categories: the rotational (DCP-R), translational (DCP-T), and hybrid (DCP-H) joints. However, little studies have been devoted to systematically investigate the biomechanical differences of dynamic cervical plates.

**Materials and methods:**

The biomechanical tests of load-deformation properties and failure modes between the SCP and DCP systems are implemented first by using the UHMWPE blocks as the vertebral specimens. The CT-based C2-C7 model simulates the strategies of cervical plate in ACDF surgery is developed with finite-element analyses. One intact, one SCP and two DCP systems are evaluated for their biomechanical properties of bone fusion and tissue responses.

**Results:**

In the situation of biomechanical test, The mean values of the five ACDSP constructs are 393.6% for construct stiffness (*p* < 0.05) and 183.0% for the first yielding load (*p* < 0.05) less than those of the SCP groups, respectively. In the situation of finite-element analysis, the rigid-induced ASD is more severe for the SCP, followed by the DCP-H, and the DCP-R is the least.

**Discussion and conclusions:**

Considering the degenerative degree of the adjacent segments and osteoporotic severity of the instrumented segments is necessary while using dynamic system. The mobility and stability of the rotational and translational joints are the key factors to the fusion rate and ASD progression. If the adjacent segments have been degenerative, the more flexible system can be adopted to compensate the constrained mobility of the ACDF segments. In the situation of the osteoporotic ACDF vertebrae, the stiffer system is recommended to avoid the cage subsidence.

## Introduction

Anterior SCP has been widely used to promote the interbody fusion and diminish the cage-loosening complications after multilevel ACDF. Theoretically, it is aimed to immobilize the ACDF segments; thus increasing fusion rate and decreasing surgery failure [1] However, the randomized control trial studies have still reported some SCP-related complications such as plate separation from bone, screw pullout, and screw breakage [2–4].{Campos, 2014 #8} Additionally, the high rigidity of the SCP potentially results in stress-shielding effect on the ACDF segments and decreases the mechanical loads crucial to effective graft fusion [5, 6]. The immobilization of ACDF segments often accelerates the progression of the ASD due to the compensated mobility and loads from the ACDF to adjacent segments [4, 7]. Consequently, some DCP designs have been developed to allow the plate-screw and plate-plate mobility and improve the aforementioned SCP drawbacks.

**Fig. 1:**
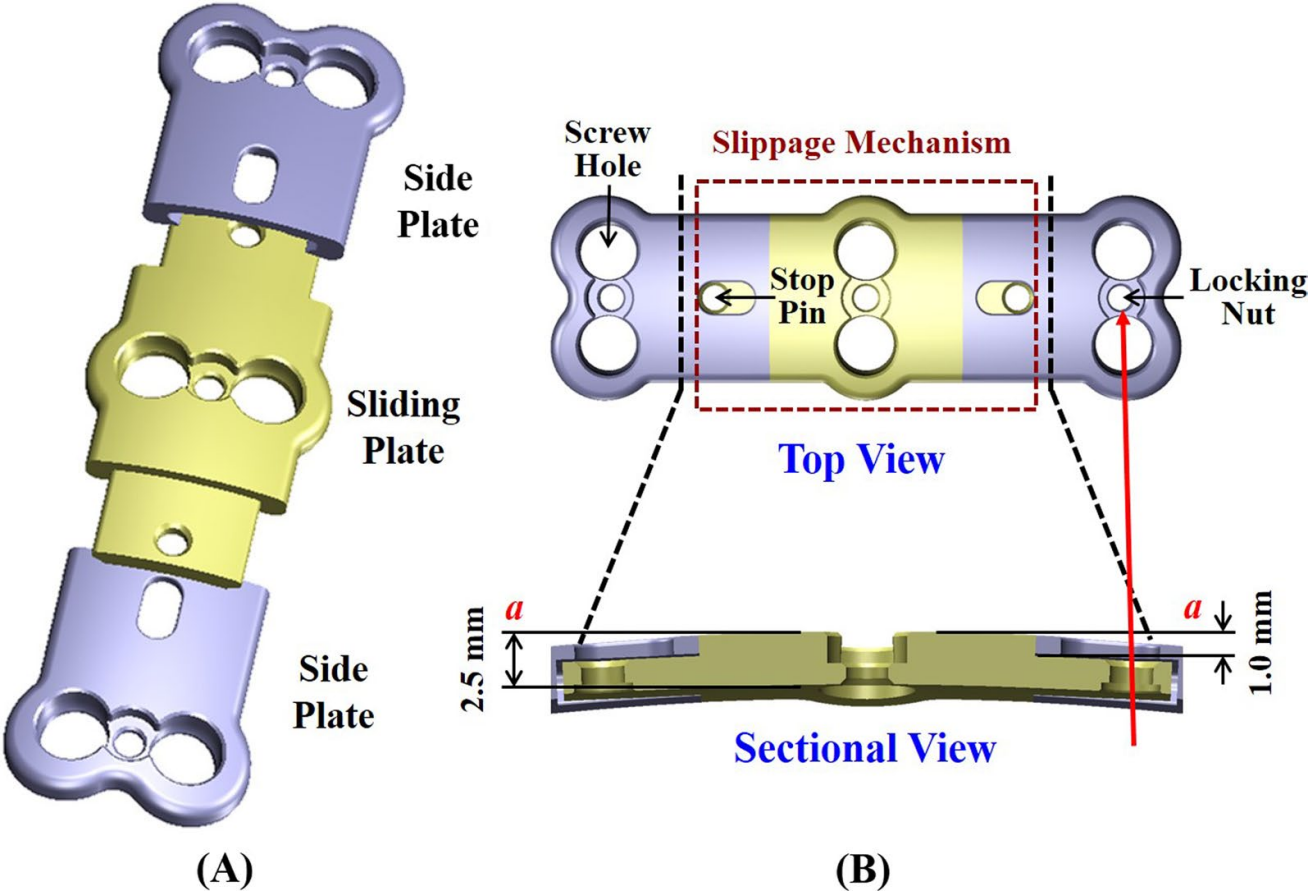
The basic components and specifications of the ACDSP plate used in this study. **A** Isometric view. The sliding plate is sandwiched between two side plates and constrained by two stop pins. **B** Top and sectional views. The plate-screw junctions are stabilized by the locking nuts. Within the slippage mechanism, the thickness of the sliding and side plates is reduced to accommodate each other

For the DCP-R design (*e.g.* Codman system: Johnson & Johnson, USA), the plate-screw joints allow the semi-constrained rotation to provide the polyaxial insertion and prevent screw back-out. For the DCP-T design (*e.g.* ACDSP system, DoubleEngine, China), the plate thickness is half reduced to form the slippage mechanism of the plate-plate joints. thus allowing translation nearly tangential to the plate profile (Fig. 1A). The rotational joints of the ACDSP system are formed by the semi-locked mechanisms of the screws and locking nuts (Fig. 1B). The plates of the another DCP-T design (*e.g.* DOC system: DepuyAcromed, USA) can translate along the linked rods. As the hybrid use of the rotational and translational joints, some DCP-H design (*e.g.* Premier system: Medtronic Sofamor Danek, USA) aims to provide the higher mobility at the screws and slot holes of the plates to the ACDF segments.

Theoretically, the characteristic differences of the rotational and translational joints affect the mobility-constraining ability (ASD problem) and load-shielding performance (cage subsidence) at the instrumented region. From the biomechanical viewpoint, the geometric discontinuity of the dynamic joints might concentrate the stress around them and induce the interfacial loosening. For the Premier system, the loss of the interfacial stability at the slot holes deteriorates the transmitted loads and induces the bone-cage subsidence across the ACDF segments. For the ACDSP and DOC systems, the tangential translation along the plate or rod profile might provide the limited flexion–extension mobility of the instrumented region and less contribute to the alleviation of the ASD problem.

This study hypothesizes that degenerative condition of the adjacent segments and bone quality of the instrumented vertebrae should be taken into consideration about surgical strategy of using DCP systems. For the adjacent segments with/without early degeneration, the long-term change in the mobility-constraining difference between the DCP systems should be carefully evaluated pre-operatively. For the poor quality of the instrumented vertebrae, the transmitted loads should be well controlled to avoid the cage subsidence. It indicates the cautious choice of the different DCP stiffness as the critical step to treat the osteoporotic patient. In literature, however, there is little study devoted to systematically investigate the short- and long-term effects of the rotational and translational mechanisms on the failure modes of the DCP joints, the transmitted loads of the ACDF segments, and the compensated responses at the adjacent segments.

This study aims to compare the biomechanical differences between the dynamic joints in terms of three effects: implant behavior (elastic stiffness of the corpectomy construct), bone fusion (intervertebral loads at the ACDF segment), and tissue degeneration (compensated motion and stress at the adjacent segments). The former one is evaluated by the biomechanical tests and the latter tows are conducted by finite-element analyses. The intact and SCP constructs are numerically chosen as the comparison baselines that represent the normal and deteriorative situations of intersegmental motion and loads. The results of the current study are correlated with the clinical and experimental results of the literature studies to provide an insight into the biomechanical differences between the SCP and DCP systems.

## Materials and methods

### Biomechanical tests

**Fig. 2:**
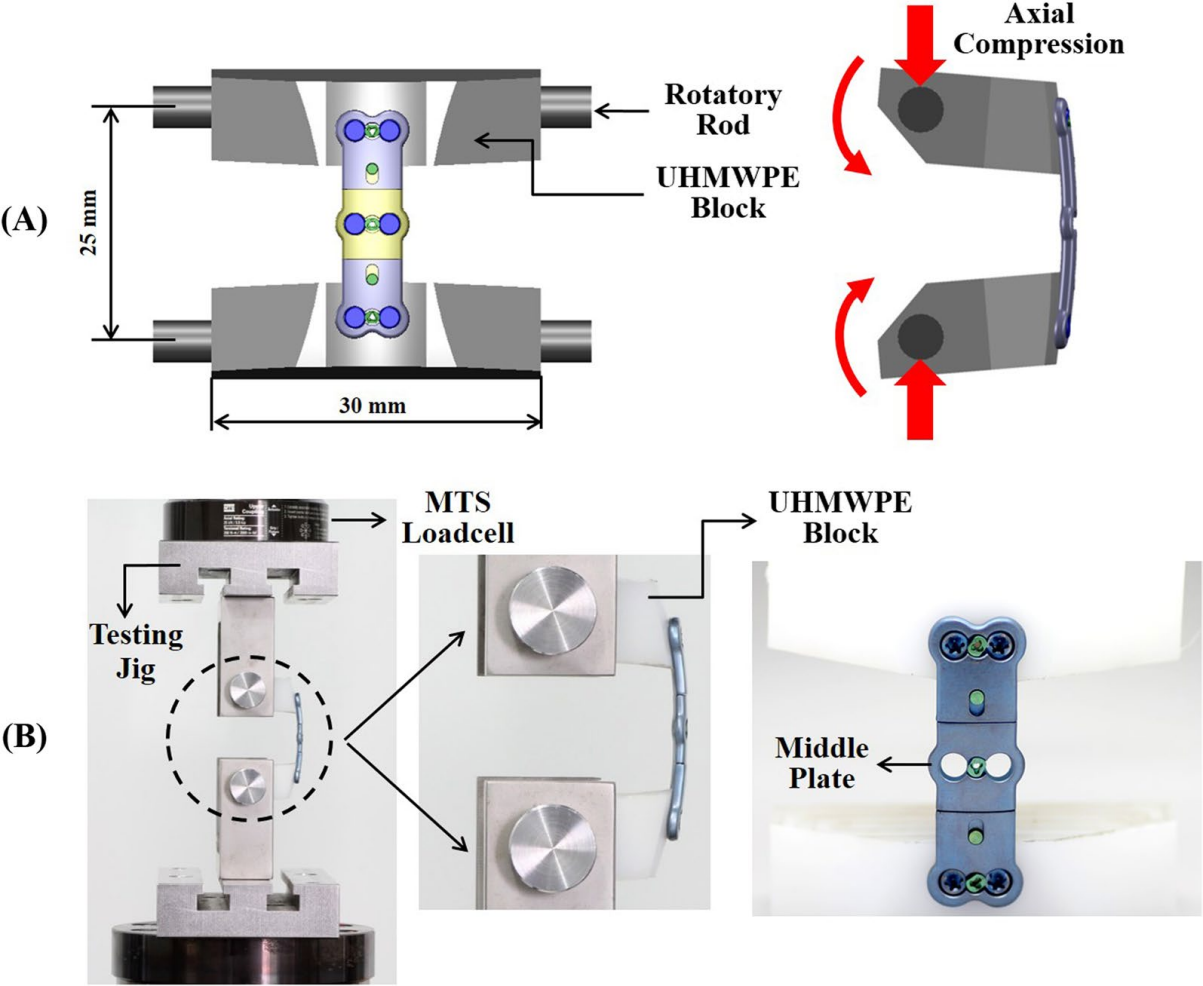
Specimens and setup of the biomechanical tests for the SCP and ACDSP specimens. **A** The titanium-based plates are fixed to the UHMWPE blocks to form a corpectomy construct that serves as the worst-case condition to the bridged plate-screw system. **B** The constructs are mounted to the testing jig that is driven by the actuator of the MTS testing system

The SCP and ACDSP specimens of the same specifications are made from the titanium alloy (Ti6Al4V) to perform the substantially equivalent comparison. For the SCP system, the screw head and the plate hole are tightly interlocked by the threads. According to the ASTM F1717 standard, two specially designed UHMWPE blocks are used as the cervical vertebrae that are spanned as a corpectomy construct to simulate the worst-case condition for stressing the anterior plates (Fig. 2A). The block-plate-screw constructs are assembled by a neurosurgeon and mounted to the testing jigs. After assembly, the single-load testes are conducted to measure the yielding loads and elastic stiffness of the SCP and ACDSP constructs (Fig. 2B). While testing, the axial compression and displacement of the corpectomy construct are recorded by the loadcell and LVDT sensors of the MTS Bionix 858 system (MTS Co., Ltd., Minneapolis, MN, USA). The control waveform of the MTS actuator is 1-Hz ramp-down function and the sampling rate of recording data is 50 Hz to plot the load–displacement curves that both yielding load and elastic stiffness can be determined. With the ramp-down waveform, the axially moving actuator of the MTS machine moves at a constant speed of 25.4 mm/min. The test is terminated when plastic deformation or implant breakage are observed with the naked eyes.

Each plate uses five specimens (n = 5) to conduct the single-load tests. No implant is reused for testing. The mean and standard deviation of the measured results are calculated and statistically analyzed. The student’s t-test with significant level (*α* = 0.05) is adopted to perform the statistical comparison between the two indices of the SCP and ACDSP constructs. The software used to compare the statistical difference is Excel ver. 2016 software (Microsoft Corporation, WA, USA).

### Finite-element analysis

**Fig. 3:**
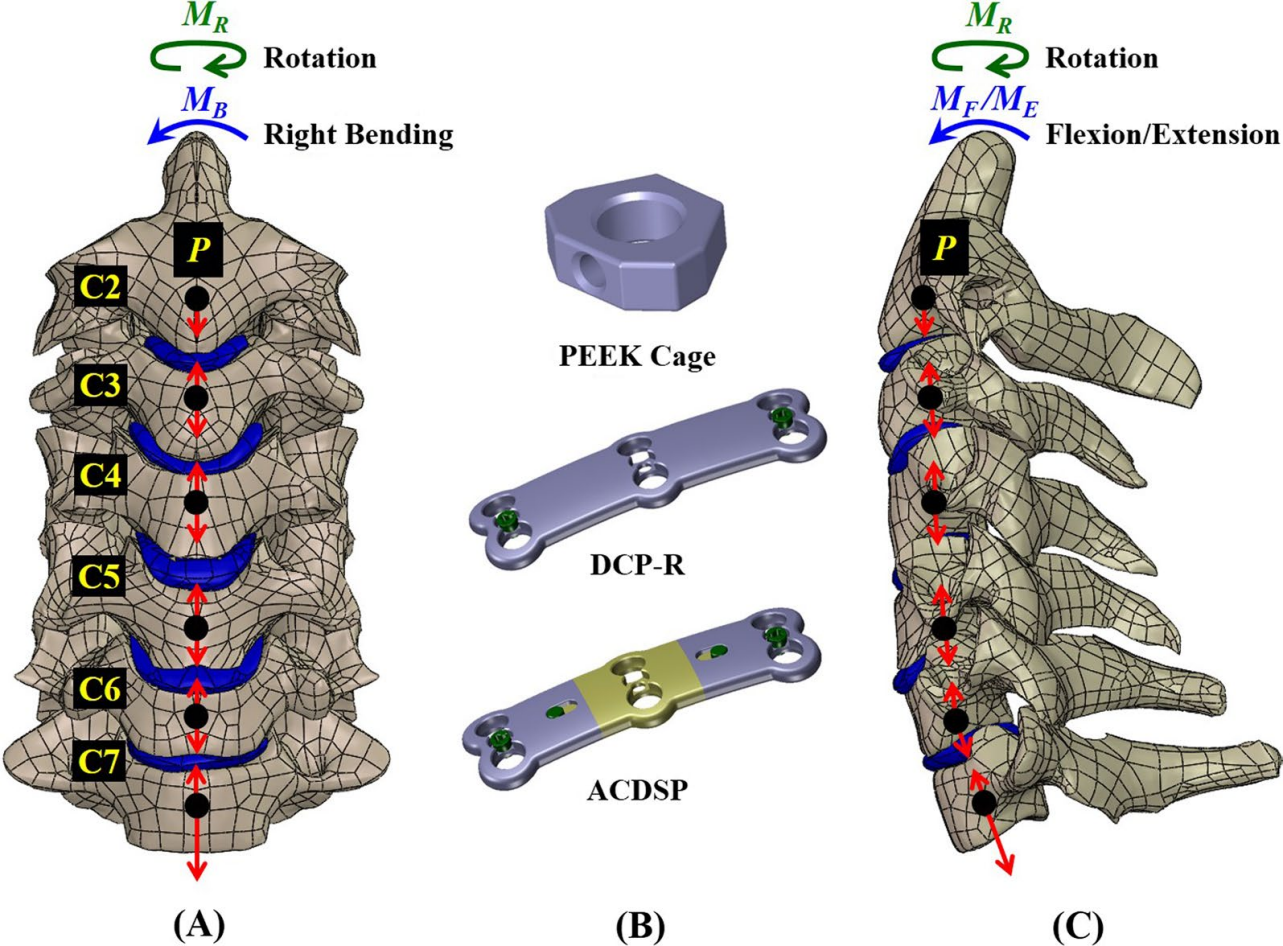
The motion of the C2-C7 cervical model is driven by follower loads (*P*) and cranial moments (*M*_*F*_, *M*_*E*_, *M*_*B*_, and *M*_*R*_) to activate flexion, extension, bending, and rotation. **A** Front view. **B** Fusion cage and fixation plates. **C** Lateral view

**Fig. 4:**
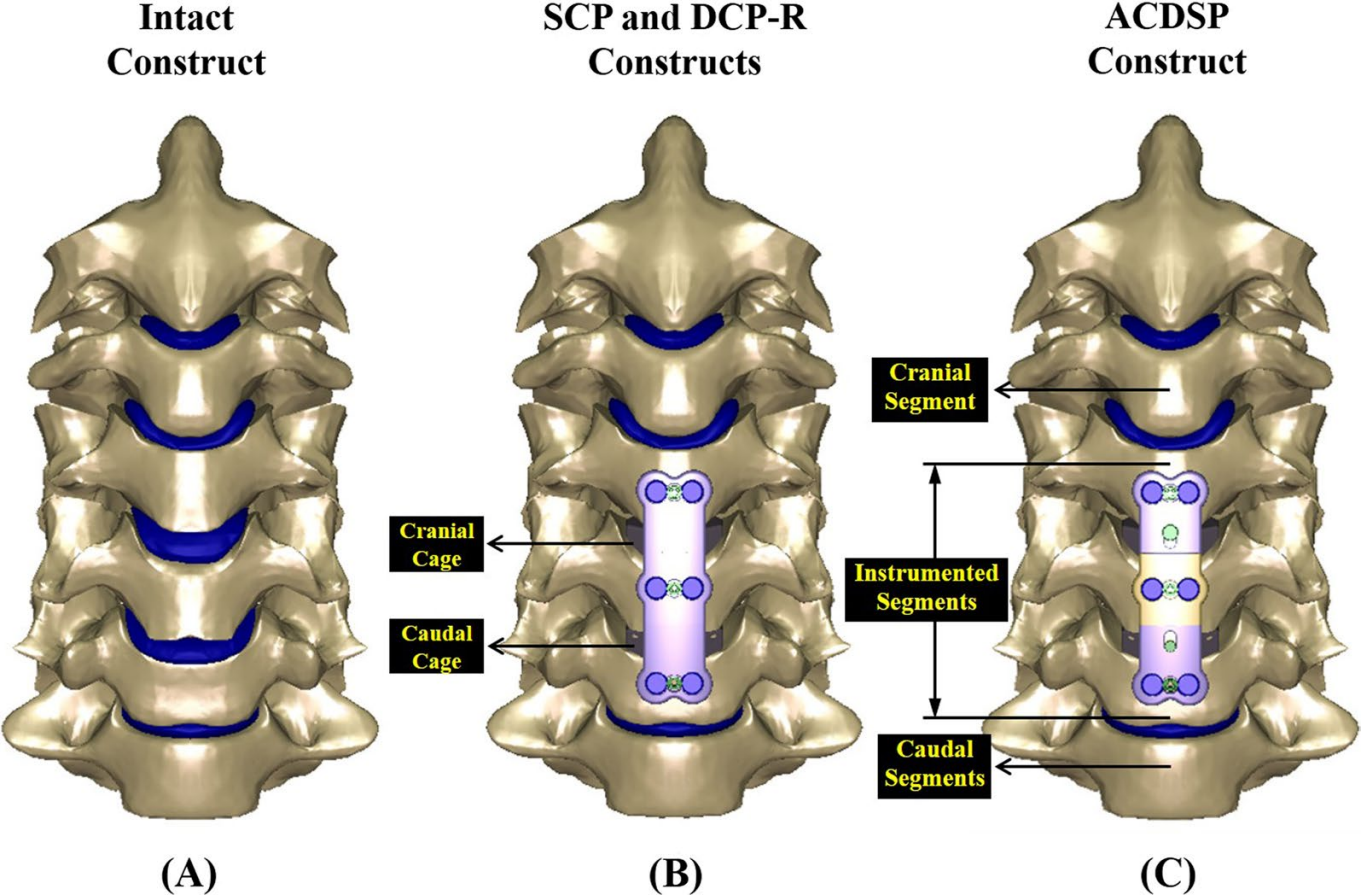
Three finite-element constructs investigated in this study. **A** Intact construct. **B** SCP and DCP-R constructs. **C** ACDSP construct

Three plate systems (SCP, DCP-R, and DCP-T) are instrumented into the cervical finite-element models. Except for the implant geometry, the constraints, loads, meshes (strategies and quality evaluation), and material properties of the bone and implants are the same and only the numbers of the meshes are different among them. The finite-element model used in this study has been validated and published by the current authors [7]. The osseoligamentous C2-C7 model consists of vertebral bones (anterior bodies, posterior elements, and endplates), intervertebral discs (annulus fibrosus and nucleus pulposus), and surrounding ligaments (Fig. 3A and C). The detailed information of the finite-element model has been described in the previous work of the current authors [7]. The basic components and specifications of the SCP and ACDSP are schematically shown in Fig. 1. Based on the intact construct, there are two types of surgical strategies investigated in this study (Fig. 4A and B and C). For the SCP and DCP constructs, the C4-C5 and C5-C6 segments are instrumented by two peek cages, followed by SCP and DCP fixation through C4-C6 segments, respectively. The peek cage applied in this study is the Cervios system (Synthes Inc., Paoli, PA, USA). The cage spikes and the threads of the plate holes and locking screws are ignored for computational competence (Fig. 3B). The placement of the anterior static and dynamic cervical plates and two peek cages are monitored by two neurosurgeons. This study uses the footings “cranial” and “caudal” to represent the different positions of two cages and adjacent (C2-C3 to C3-C4 and C6-C7) segments, individually (Fig. 4B and C). The three-dimensional models of all implants are developed by the software SolidWorks Ed. 2018 (SolidWorks Corporation, Concord, MA, USA).

The follower loads (*P* = 73.6 N) and concentrated moments (*M*_*i*_ = 1.0 Nm, *i* = *F*, *E*, *B*, *R*) are applied to simulate flexion, extension, lateral bending, and axial rotation of the cervical column that the bottom surface of the C7 vertebral body is fully constrained (Fig. 3). The follower loads (73.6 N) are used to simulate the muscular contractions and stabilize the cervical column. The concentrated moment are driven from head weight and muscular contractions and applied at the cervical top. Using the displacement-controlled method the criterion for controlling the same motion of cervical constructs is adapted as a reasonable approach to evaluate the implant-induced effects on the instrumented and adjacent segments.

There are three types of static and dynamic plates simulated in this study: SCP, DCP-R, and ACDSP systems (Fig. 4 B and C). The interfaces between facet joints, bone-plate interfaces, plate/plate joints, plate/nut joints, and screw/nut joints are modeled as the surface-to-surface contact elements which allow separation and slippage and exclude the friction. The bone-screw interfaces are assumed bonded to simulate perfectly bony purchase. The elastically constrained interaction between the locking nuts, screw heads, and plate holes is determined by finite-element simulation. For the SCP system, the plate-screw joints are assumed to fully bonded.

All implant materials are assumed to have linearly elastic, homogeneous, and isotropic properties throughout. The calculated von Mises stresses of all implants are compared with the yielding strength of the corresponding material to validate the assumption of linear elasticity. The strategies of mesh control and energy convergence have been described in the previous study of the current authors. Using aspect ratio and the Jacobian check, the quality of all elements are monitored to avoid sharp discontinuities and unrealistically high stress concentrations. The nonlinear algorithm with large-deformation formula and direct-sparse solver is used by the software Simulation Ed. 2018 (SolidWorks Corporation, Concord, MA, USA). There are two types of the numerical indices used in this study: compensated motion and stress at the adjacent segments and intersegmental loads across the ACDF and adjacent segments. The former is the angles and stresses of the cranial and caudal discs to provide the ASD information due to instrumentation. The latter can reveal the stiffness-induced differences in the cage fusion between the one SCP and two DCP systems.

## Results

### Biomechanical tests

**Fig. 5:**
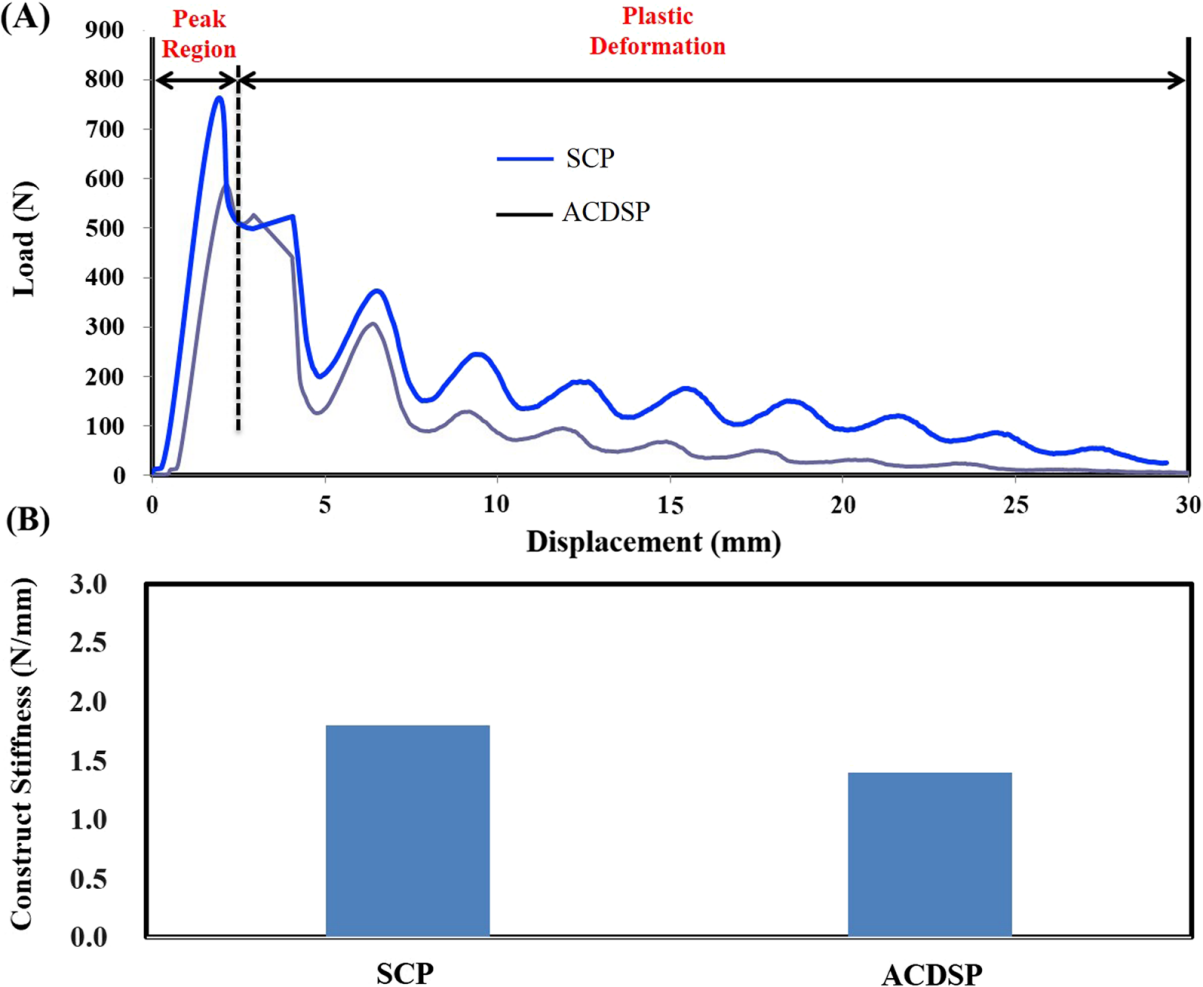
The single-load results of two SCP and ACDSP constructs. **A** The typical pattern of the load-deformation curves during axial compression. **B** The statistical results of the construct stiffness for the SCP (12.6, 14.5, 13.2, 14.2, and 14.5) and ACDSP (2.5, 2.9, 3.0, 2.7, 2.9)

The load-deformation curves of the single-load tests are plotted to show the elastic and plastic regions of the tested constructs (Fig. 5A). The plastic deformation of the SCP constructs is concentrated at the plate-screw interfaces that are the sites of the geometric discontinuity. Similarly, the plate-screw-nut joints of the ACDSP constructs are consistently the first yielding region for the plastic deformation of the locking nuts. After plate-screw-nut yielding, the stable contact still remains at the plate-plate joint that shows the stiffer resistance to the axial compression. The load-deformation curve of the SCP construct is remarkably above the that of the ACDSP. The SCP curve show the smoother profile than the ACDSP. The statistical information about construct stiffness are calculated to compare the structural differences between the SCP and ACDSP (Fig. 5B). The mean values of the five ACDSP constructs are 393.6% for construct stiffness (*p* < 0.05) and 183.0% for the first yielding load (*p* < 0.05) less than those of the SCP groups, respectively.

### Finite-element Analyses

**Fig. 6:**
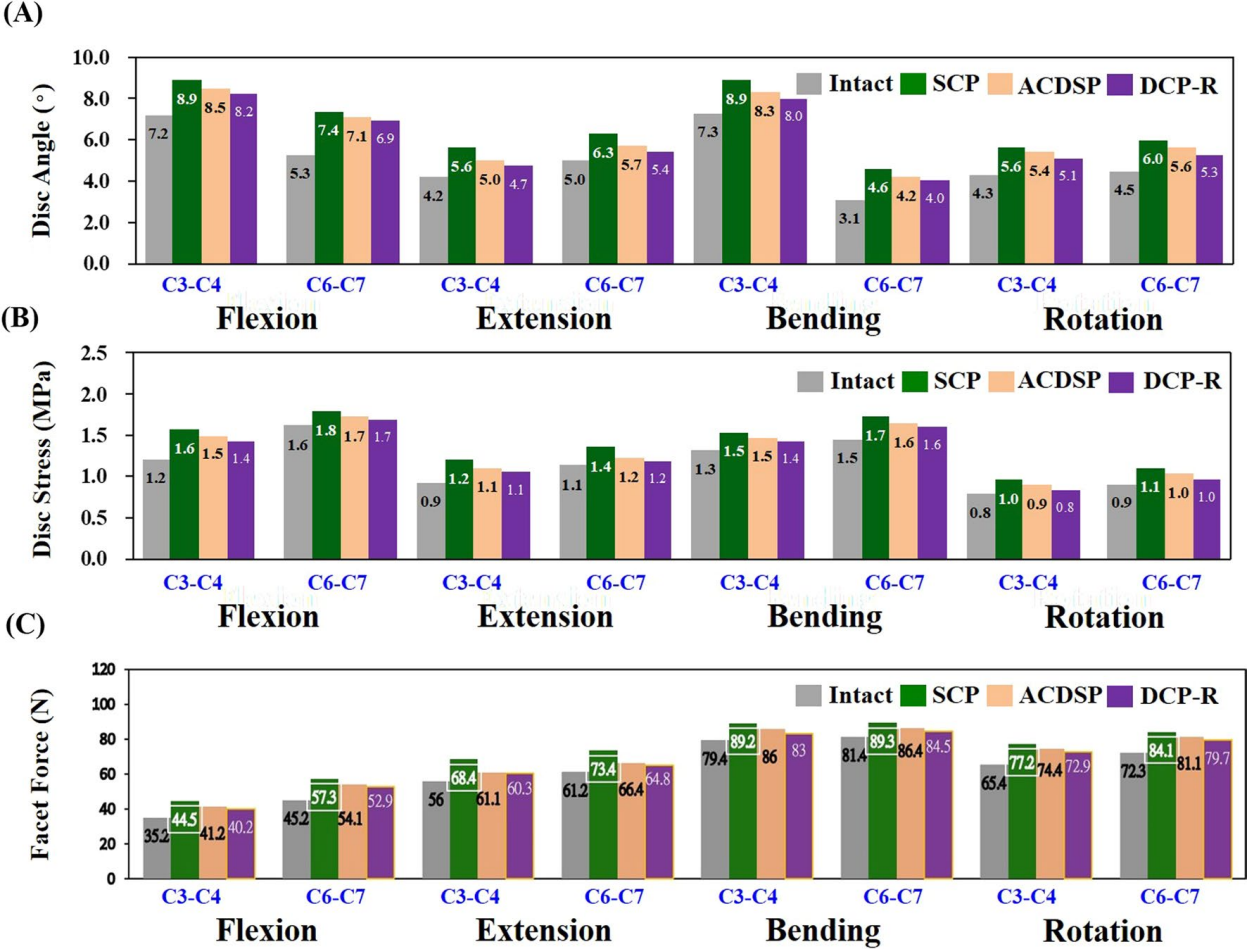
The predicted kinematic and mechanical results of the adjacent segments. **A** Disc angles. **B** Disc stresses. **C** Facet forces

The stiffness-raising effects of one SCP and two DCP fixation are firstly compared in terms of adjacent tissue responses (Fig. 6). Compared with the intact construct, the SCP increases the disc angle, discs stress, and facet force at the C3-C4 (C6-C7) segment by 23.6% (39.6%), 29.8% (12.5%), and 26.4% (26.8%) for flexion, 33.3% (26%), 30.4% (27.3%), and 22.1% (19.9%) for extension, 21.9% (48.4%), 15.4% (13.3%), and 12.3% (9.7%) for bending, and 30.2% (33.3%), 25.0% (22.2%), and 18.0% (16.3%) for rotation, respectively. For the ACDSP, the aforementioned results are increased as 18.1% (33.9%), 25.0% (6.2%), and 17.0% (19.7%) for flexion, 19.0% (14%), 22.2% (9.1%), and 19.7% (8.5%) for extension, 13.7% (35.5%), 15.4% (6.7%), and 8.3% (6.1%) for bending, and 25.6% (24.4%), 12.5% (11.1%), and 13.8% (12.2%) for rotation, respectively, but these results are less than results of SCP. Compared with the ACDSP, the results of the DCP-R are raised up to 13.9% (30.2%), 16.7% (6.3%), and 14.2% (17.0%) for flexion, 11.9% (8.0%), 22.2% (9.1%), and 7.7% (5.9%) for extension, 9.6% (29%), 7.7% (6.7%), and 4.5% (3.8%) for bending, and 18.6% (17.8%), 10.1% (11.1%), and 11.5% (10.2%) for rotation, respectively (Table 1).

**Table 1:** Predicted kinematic and mechanical differences

	ACDSP		DCP-R		ACDSP		DCP-R	
	C3-4				C6-7			
*Flexion*								
Disc angle	18.1%	− 5.5%	13.9%	− 9.7%	33.9%	− 5.7%	30.2%	− 9.4%
Disc stress	25.0%	− 4.8%	16.7%	− 13.1%	6.2%	− 6.3%	6.3%	− 6.2%
Facet force	17.0%	− 9.4%	14.2%	− 12.2%	19.7%	− 7.1%	17.0%	− 9.8%
*Extension*								
Disc angle	19.0%	− 14.3%	11.9%	− 21.4%	14.0%	− 12.0%	8.0%	− 18.0%
Disc stress	22.2%	− 8.2%	22.2%	− 8.2%	9.1%	− 18.2%	9.1%	− 18.2%
Facet force	19.7%	− 2.4%	7.7%	− 14.4%	8.5%	− 11.4%	5.9%	− 13.8%
*Bending*								
Disc angle	13.7%	− 8.2%	9.6%	− 12.3%	35.5%	− 12.9%	29.0%	− 19.4%
Disc stress	15.4%	0.0%	7.7%	− 7.7%	6.7%	− 6.6%	6.7%	− 6.6%
Facet force	8.3%	− 4.0%	4.5%	− 7.8%	6.1%	− 3.6%	3.8%	− 5.9%
*Rotation*								
Disc angle	25.6%	− 4.6%	18.6%	− 11.6%	24.4%	− 8.9%	17.8%	− 15.5%
Disc stress	12.5%	− 12.5%	10.1%	− 14.9%	11.1%	− 11.1%	11.1%	− 11.1%
Facet force	13.8%	− 4.2%	11.5%	− 6.5%	12.2%	− 4.1%	10.2%	− 6.1%

The comparison between SCP and two DCPs using SCP as a benchmark showed that DCP-R had a higher decrease in disc angle, disc stress and facet force than ACDSP

Between the SCP and two DCP fixation, the kinematic and mechanical differences at the C3-C4 segment are 4.5%, 4.6%, and 8.0% for flexion, 10.7%, 8.3%, and 10.7% for extension, 6.7%, 6.2%, and 3.6% for bending, and 3.6%, 7.3%, and 3.4% for rotation, respectively. In general, cervical extension (about 9.9%) shows the more significant differences in compensated motion and stress than the others (about 5.3%).

**Fig. 7:**
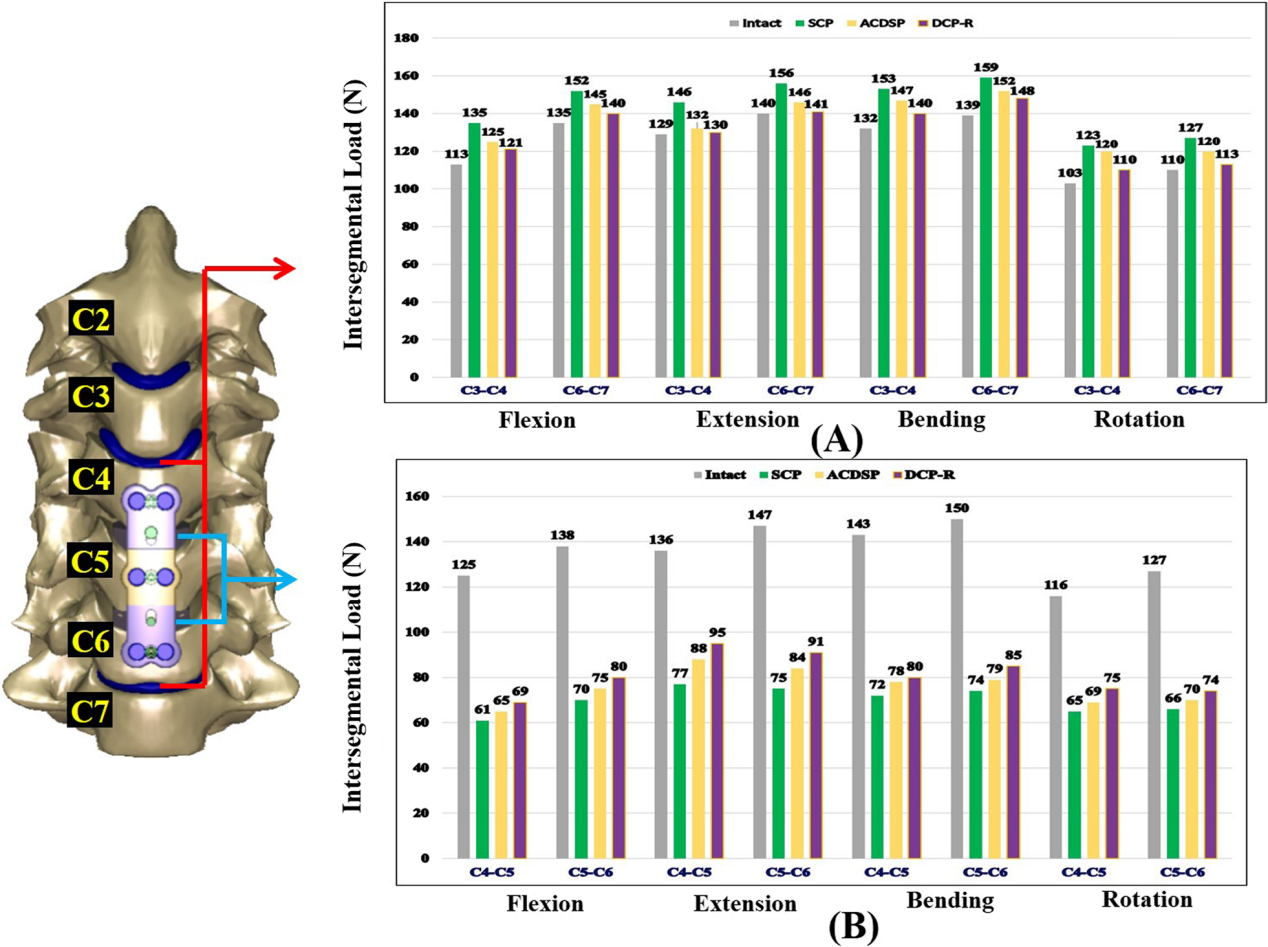
The intersegmental loads are used as the indices to evaluate fusion rate and ASD progression. **A** and **B** The intersegmental loads of the adjacent (C3/C4 and C6/C7) and instrumented (C4/C5 and C5/C6) segments, respectively

The interbody loads through the intact and ACDF segments provide the fusion-related information (Fig. 7A). Compared with the intact construct, the intersegmental loads of the SCP construct in cervical flexion are deteriorated by 19.5% for the cranial segment and 12.6% for the caudal segment, respectively. For cervical extension, bending, and rotation, the aforementioned differences are 13.2%, 15.9%, and 19.4% for the cranial segment and 11.4%, 14.4%, and 15.5% for the caudal segment, respectively. Using the ACDSP construct, the increased loads at the cranial and caudal segments can be suppressed as 10.6% and 7.4% for flexion, 2.3% and 4.3% for extension, 11.4% and 9.4% for bending, and 16.5 and 9.1% for rotation, higher than those of the intact construct, respectively. In the DCP-R construct, the compensated loads at the cranial and caudal segments are reduced by 7.1% and 3.7% for flexion, 0.8% and 0.7% for extension, 6.1% and 6.5% for bending, and 6.8% and 2.7% for rotation, respectively.

Within the instrumented region, the intersegmental loads of the flexed SCP construct are 51.2% at the C4-C5 segment and 49.3% at the C5-C6 segment less than those of the intact construct, respectively (Fig. 7B). For cervical extension, bending, and rotation, the load decreases are 43.4%, 49.7%, and 44.0% at the C4-C5 segment and 49.0%, 50.7%, and 48.0% at the C5-C6 segment, respectively. For the ACDSP construct, the weaken stiffness of the slippage mechanism makes the aforementioned differences as 48.0% and 45.7% for flexion, 35.3% and 42.9% for extension, 45.5% and 47.3% for bending, and 40.5% and 44.9% for rotation. In general, the SCP fixation shields the C4-C5 and C5-C6 loads 3.2% and 3.6% for flexion, 8.1% and 6.1% for extension, 4.2% and 3.4% for bending, and 3.5% and 3.1% for rotation compared with ACDSP fixation (Table 2).

**Table 2:** Difference of intersegmental loads

	ACDSP		DCP-R		ACDSP		DCP-R	
	C4-5				C5-6			
Flexion								
Intersegmental loads	↓48.0%	+ 3.2%	↓44.8%	+ 6.4%	↓45.7%	+ 3.6%	↓42.0%	+ 7.3%
Extension								
Intersegmental loads	↓35.3%	+ 8.1%	↓30.1%	+ 13.3%	↓42.9%	+ 6.1%	↓38.1%	+ 10.9%
Bending								
Intersegmental loads	↓45.5%	+ 4.2%	↓44.1%	+ 5.6%	↓47.3%	+ 3.4%	↓43.3%	+ 7.4%
Rotation								
Intersegmental loads	↓40.5%	+ 3.5%	↓35.3%	+ 8.7%	↓44.9%	+ 3.1%	↓41.7%	+ 6.3%

The comparison between SCP and two DCPs using SCP as a benchmark showed that DCP-R shields the C4-5 and C5-6 intersegmental loads less than ACDSP. The stiffer SCP can remarkably shield the intersegmental loads

## Discussions

### Biomechanical tests

With the locking joints, the SCP constructs show the significantly higher stiffness and yielding load than the ACDSP (Fig. 5A). Compared with the stiffer plate-plate joints, the local yielding of the ACDSP constructs consistently occurs at the plate-screw-nut joints until the tests are terminated (Fig. 5B). This can be accounted for the more stable mechanism of the plate-plate than plate-screw-nut joint that the locking nuts are structurally weaker than the interfaces of the side-slide plates (Fig. 1A). This indicates that the plastic deformation of the plate-screw-nut joints dominates the mechanical properties of the ACDSP system. However, this cannot be directly applied to Premier system that the allows higher mobility by means of screw slippage within the slot hole [8]. This further reveals the design information that the mobility and stability of the rotational joints should be arranged to comparable to the translational joints.

### Finite-element analyses

In general, the compensated disc angles, disc stresses, and facet forces of the static and dynamic fixations consistently show more severe at the cranial than caudal segments (Fig. 5). These findings are similar to the clinical results that show the ASD progression at the cranial segments [9]. The rigid-induced ASD is more severe for the SCP, followed by the DCP-H, and the DCP-R is the least. The reason can be attributed to the highest mobility at the plate/screw joints of the DCP-R than the counterparts. Except for extension, the predicted ACDSP DCP-H results show the quite minor contribution to suppress the ASD progression (Fig. 6A). This can be explained by the fact that the plate-plate joints only provide the tangential translation along the plate profile (line aa) (Fig. 3B). This indicates that the plate-plate mobility of the ACDSP DCP-H is nearly orthogonal to cervical flexion, bending, and rotation. Except for the extension, this makes the constrained mobility of the ACDSP DCP-H construct approximately comparable to that of the SCP.

Among the existing DCP designs (*e.g.* ACDSP, DOC, and Premier systems), the degree-of-freedom of the plate-plate joints and mobility-activating sequence of the rotational and translational joints are quite different. Except for extension, the translational joints of the ACDSP system is nearly immobile and stiffer than the rotational joints; thus the local slippage and even plastic deformation concentrates at the plate-screw joints (Fig. 7A). This induces the activation of the rotational joints prior to the translational joints. For the Premier system, however, the translation of the screws along the slot-holes begins before the rotation of the plate-screw joints and is less constrained as compared with the ACDSP design. The other studies have consistently reported that the Premier system provides the higher translational mobility than the other DCP-R counterparts (C-tek system, Biomet, USA and ATLANTIS system, Medtronic Sofamor Danek, USA) [8, 10]. This indicates that the predicted ACDSP DCP-H results cannot be directly applied to the other systems. If the translational joints of the DCP-H can initially provide the higher mobility than the rotational joints, the load-sharing percentage of the bone-cage interfaces and the biomechanical compensation at the adjacent segments definitely differ from the results of the ACDSP systems. For example, the intersegmental mobility (C4-C5 and C5-C6) instrumented by the ACDSP DCP-H is predicted as higher reduction than by the DCP-R (Fig. 5A). However, the Brodke et al*.* study had reported the different results for the Premier DCP-H system [8].

Within the adjacent C3-C4 and C6-C7 segments, the increase of the intersegmental load is the highest for the SCP, followed by the DCP-T, and the DCP-R is the least (Fig. 6B). This can be explained by the fact that the more rigid constraint of the fixator induces the higher biomechanical compensation at the adjacent segments. The stiffer SCP shows the higher load-transferring ability to the instrumented segments than its counterparts (Fig. 6C). Theoretically, the decreased stiffness and/or increased mobility suppresses the load-transferring capacity of the DCP implant and transfers the higher load to the interbody cage and bone graft [11]. This predicts the higher intersegmental loads of the instrumented segments by the DCP-R, followed by the DCP-T and SCP. The predicted results of the intersegmental loads are consistent with the testing results of the Brodke et al. study [8]. Using seven human cadavers as specimens, their testing reports show the load-sharing percentage (= 58%) of the DCP-H less than that (68%) of the DCP-R without statistical significance. In the clinical study, the cervical height decreases in DCP-H group more than in SCP group postoperatively. This is consistent with higher subsidence rate of fusion cage in DCP-H group than in SCP group [12]. 

**Fig. 8:**
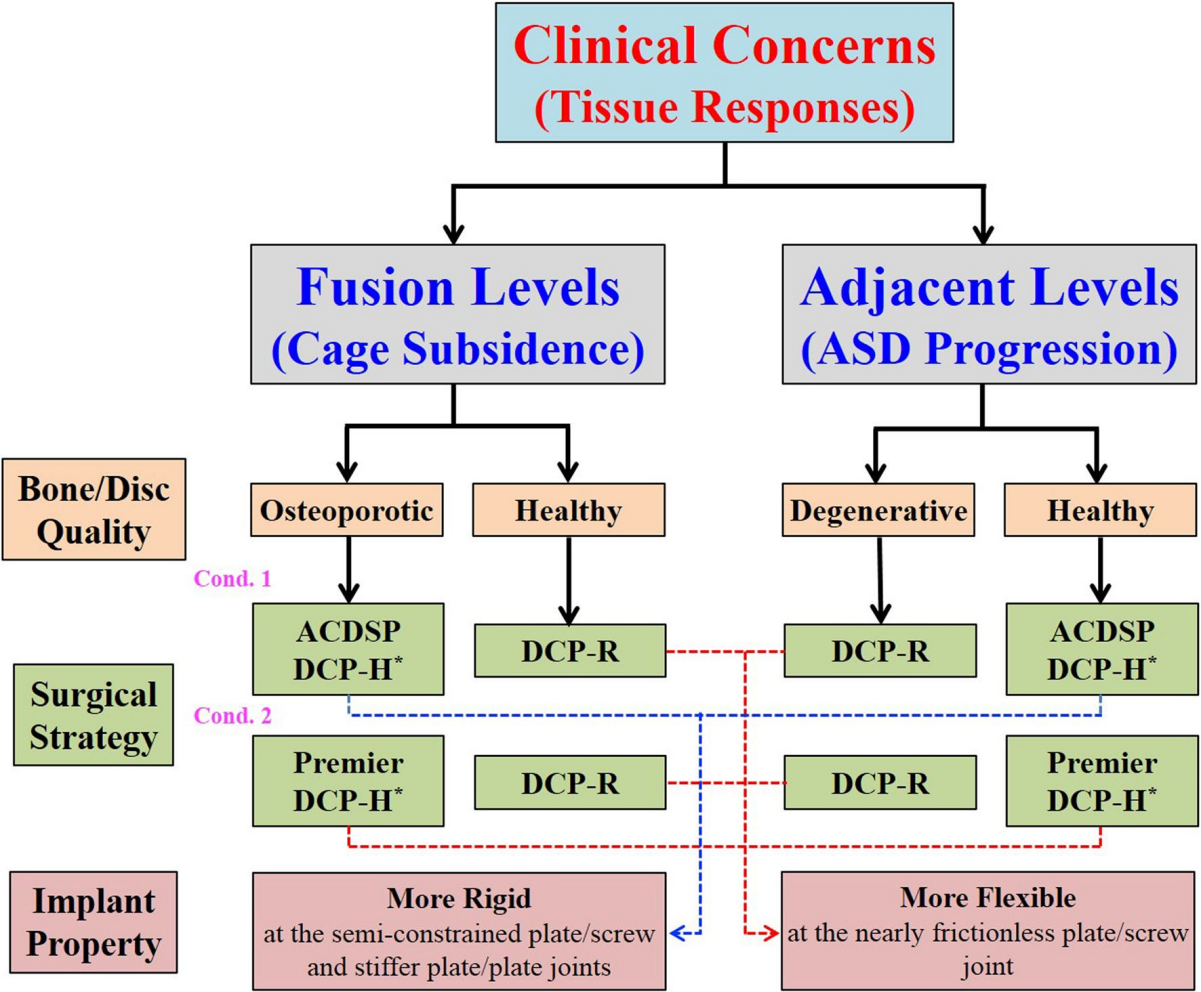
The flowchart to illustrate the surgical strategies of using DCP systems in terms of tissue responses (cage subsidence and ASD progression) and the bone/disc quality at the ACDF segments. There are two conditions of using DCP-H strategies: ACDSP and Premier systems. The reasons of surgical priority are attributed to the mobility and stability of the DCP joints

Some papers indicate that implant subsidence can be significantly related to the value of osteoporosis [13, 14]. Higher rigidity of the static plate may accelerate the progression of the adjacent segment degeneration [15]. The strategy priority of using DCP-R and DCP-H is schematically illustrated by degenerative extent of the adjacent segments and bone quality of the ACDF segments (Fig. 8). Two conditions of the DCP-H joint should be cautiously evaluated to choose which implant is used. The first is the degenerative degree of the adjacent segments. Initial rotation and plastic deformation of the plate-screw joints makes the DCP-R to behave as a provider of the limited mobility to the adjacent segments. The second is the bone quality of the instrumented segments. The plate-plate joints of the DCP-H are stiffer than the plate-screw joints of the DCP-R. This makes the DCP-H as the preferred strategy to stabilize the osteoporotic segments. For the Premier system, however, the higher mobility of the screw and slot-holes makes the bone-cage subsidence as the potential concern of the using DCP-H [8]. This is not the situation of the ACDSP system.

### Limitations of this study

The scenario of this study is designed to simulate biomechanical characteristics of the cervical plates before interbody fusion. This allows the occurrence of bone-cage separation and makes the tissue- and implant-related properties of the SCP and DCP joints to be more clarified. Consequently, there are four limitations inherent in this study. The first two are the assumptions of no intervertebral fusion at the ACDF segments and no loosening ay the bone-screw interfaces. These are the just post-operative situation that the implant-induced effects on the tissue responses and implant behaviors can be explicitly clarified. The third is the results of the single-load tests that provide only short-term information of the bone-implant construct. The resistance of the plate-plate and plate-screw joints to the cyclic loads is not evaluated in this study. The final is the use of the corpectomy model that the load-transferring ability of the sandwiched cages is underestimated. The testing results potentially overestimate the implant-induced effects on the tissues and implant themselves.

In conclusion, the degenerative and osteoporotic degree of the adjacent and instrumented segments should be evaluated prior to use dynamic system. While choosing the dynamic system, the design mechanisms of the rotational and translational joints are the key factors to determine the mobility and stability. If the adjacent segments have been degenerative, the more flexible system can be adopted to compensate the constrained mobility of the ACDF segments. In the situation of the osteoporotic ACDF vertebrae, the stiffer system is recommended to avoid the cage subsidence.

## Data Availability

The datasets used and/or analysed during the current study are available from the corresponding author on reasonable request.

## Abbreviations

SCP: Static cervical plate
DCP: Dynamic cervical plate
ACDF: Anterior cervical discectomy and fusion
ASD: Adjacent segment degeneration
DCP-R: Rotational joint of DCP
DCP-T: Translational joint of DCP
DCP-H: Hybrid joint of DCP
